# Supplementary material for: Water, Sanitation and Hygiene in Schools in Low- and Middle-Income Countries: A Systematic Review and Implications for the COVID-19 Pandemic
Source: Int J Environ Res Public Health. 2022 Mar 7;19(5):3124. doi: 10.3390/ijerph19053124 (PMC8910349; doi:10.3390/ijerph19053124)
Supplement: Supplementary file 1 [file ijerph-19-03124-s001.zip › Supplementary Material S1_Search Strategy.pdf]

Water, sanitation and hygiene in schools in low- and middle-income countries and its implications for COVID-19 pandemic: A systematic review

Kasandra I.H.M Poague, Justine Blanford and Carmen Anthonj

Supplementary Material S1: Search Strategy:

	**Search terms (PubMed, Web of Science, Scopus and *Literatura Latino-Americana e do Caribe em Ciências da Saúde* - LILACS)**
**Concept 1:** Water; sanitation and hygiene *means truncation	Hygiene; Hand disinfection; Handwashing; Hand washing; Hand-washing; Toilet*; Bathroom*; Latrine; Sewage; Waste water; Waste-water; Wastewater; Septic Tank; Sanitation; Water; Drinking-water; WASH; Menstruation; Menstrual;
**Concept 2:** Schools and students *means truncation	School*; Pre-School*; Preschool*; Student*; Schoolchild*; School child; School age; School-age;
**Concept 3:** Low-and middle-income countries *means truncation	Developing countries; Third world countr*; Third-word countr*; Less developed countr*; Less-Developed countr*; Low-income countr*; Low income countr*; Middle-income countr*; Middle income countr*; LMIC*;
**Mesh Terms**	Hand hygiene; Hand disinfection; Toilet facilities; Bathroom equipment; Sewage; Drinking water; Water supply; Menstruation; Menstrual hygiene products; Schools; Students; Developing countries;
**DeCs Terms**	Higiene das mãos/Hand hygiene/Higiene de las manos; Desinfecção das mãos/Hand disinfeccion/ Desinfección de las manos; Toaletes/Toilet facilities/Cuartos de baños; Aparelho sanitário/Bathroom equipments/Aparatos sanitários; Esgoto/Sewage/Aguas del acantarillado; Abastecimento de água/Water supply/Abastecimiento de agua; Água potável/Drinking water/Agua potable; Produtos de higiene menstrual/Menstrual hygiene products/Productos para la higiene menstrual; Menstruação/Menstruation/ Menstruación; Acadêmicas/Schools/Instituciones académicas; Estudantes/Students/Estudiantes; Países em desenvolvimento/Developing countries/Países em desarrollo;
	**Search terms (African Journals Online – AJOL)**
**Concept 1:** Water; sanitation and hygiene	Hygiene; Water; Sanitation; Menstruation; Toilet; Bathroom; Sewage; Latrine;
**Concept 2:** Schools and students	School; Student;
**Concept 3:** Low-and middle-income countries	LMIC; Developing countries;
